# Landscape as a Model: The Importance of Geometry

**DOI:** 10.1371/journal.pcbi.0030200

**Published:** 2007-10-26

**Authors:** E. Penelope Holland, James N Aegerter, Calvin Dytham, Graham C Smith

**Affiliations:** 1 Central Science Laboratory, Sand Hutton, York, United Kingdom; 2 Department of Biology, University of York, York, United Kingdom; 3 Plant Ecology and Nature Conservation, University of Potsdam, Potsdam, Germany; University of Newcastle upon Tyne, United Kingdom

## Abstract

In all models, but especially in those used to predict uncertain processes (e.g., climate change and nonnative species establishment), it is important to identify and remove any sources of bias that may confound results. This is critical in models designed to help support decisionmaking. The geometry used to represent virtual landscapes in spatially explicit models is a potential source of bias. The majority of spatial models use regular square geometry, although regular hexagonal landscapes have also been used. However, there are other ways in which space can be represented in spatially explicit models. For the first time, we explicitly compare the range of alternative geometries available to the modeller, and present a mechanism by which uncertainty in the representation of landscapes can be incorporated. We test how geometry can affect cell-to-cell movement across homogeneous virtual landscapes and compare regular geometries with a suite of irregular mosaics. We show that regular geometries have the potential to systematically bias the direction and distance of movement, whereas even individual instances of landscapes with irregular geometry do not. We also examine how geometry can affect the gross representation of real-world landscapes, and again show that individual instances of regular geometries will always create qualitative and quantitative errors. These can be reduced by the use of multiple randomized instances, though this still creates scale-dependent biases. In contrast, virtual landscapes formed using irregular geometries can represent complex real-world landscapes without error. We found that the potential for bias caused by regular geometries can be effectively eliminated by subdividing virtual landscapes using irregular geometry. The use of irregular geometry appears to offer spatial modellers other potential advantages, which are as yet underdeveloped. We recommend their use in all spatially explicit models, but especially for predictive models that are used in decisionmaking.

## Author Summary

Many different areas of science try to simulate and predict (model) how processes act across virtual landscapes. Sometimes these models are abstract, but often they are based on real-world landscapes and are used to make real-world planning or management decisions. We considered two separate issues: how movement occurs across landscapes and how uncertainty in spatial data can be represented in the model. Most studies represent the landscape using regular geometries (e.g., squares and hexagons), but we generated landscapes of irregular shapes. We tested and compared how the shapes that make up a landscape affected cell-to-cell movement across it. All of the virtual landscapes formed with regular geometries had the potential to bias the direction and distance of movement. Those formed with irregular geometry did not. We have also shown that describing whole real-world landscapes with regular geometries will lead to errors and bias, whereas virtual landscapes formed with irregular geometries are free from both. We recommend the use of multiple versions of virtual landscapes formed using irregular geometries for all spatially explicit models as a way of minimizing this source of bias and error; this is especially relevant in predictive models (e.g., climate change) that are difficult to test and are designed to help make decisions.

## Introduction

The focus of this study is spatially explicit predictive models designed to support decisionmaking (e.g., population establishment and spread, climate change, and flood risk), which should have reliable, probabilistic, and mappable results. In cases in which there are few relevant validation data (e.g., nonnative species and climate change), the model cannot be calibrated statistically, and it is therefore important that biases and uncertainties are dealt with explicitly so that confidence can be placed in the results. Uncertainty may surround all components of a model (e.g., input data and processes), but bias by definition usually results from the way that processes are implemented in the model. In this study, we explored how spatial structure can be a source of bias, and present an approach that allows uncertain landscape data to be incorporated into model output with minimal bias.

There are many different landscape models in the literature (see [1] for a recent and comprehensive list), all of which allow a process (population) model to interrogate explicit locations or regions of space, and choosing the most appropriate landscape model for the study in hand is important [1]. We focus on the use of cells in a mosaic-based model [2] to represent processes in space, which requires the subdivision of space into a tessellation of discrete, internally homogeneous patches within which a process occurs. Although this is an elegant, abstract concept, the use of cells to represent uncertain spatial processes is often desirable in real-world applications. First, some information is better represented by an areal unit than by a point location (e.g., water), whereas other information is considered to conceptually occupy an area of real space defined by its boundary (e.g., an animal social group). Second, the limited understanding of many of these systems requires us to model at the scale for which most is known (e.g., the behaviour of individuals within a social group of animals is often not well-understood, whereas the size, productivity, or spatial description of the whole population may be simpler to study and is well-described). Third, the raw data used to describe the landscape (e.g., satellite and aerial photography) are subject to errors and uncertainties. By modelling processes at scales significantly larger than that of the underlying data, these problems become statistically tractable (e.g., Land Cover 2000 [3]). Although in many such models, the attribute values of cells are directly calculable from habitat or geographical data (e.g., vegetation type), here we use conceptually abstract and attribute-free cells in order to consider only geometry (specifically, shape) and neighbourhood (number and arrangement of adjacent, interacting patches) in a homogeneous landscape.

In this study, we have used population modelling concepts to demonstrate the potential for bias in cell-to-cell movement of information (e.g., individuals) resulting from the geometry of a mosaic virtual landscape. Population models predominately use raster virtual landscapes, and the description of home ranges or social groups with single squares (e.g., [4–6]) or a square arrangement (e.g., [7]) is not unusual. Cell-to-cell movement is implemented using either von Neumann (e.g., [8]) or Moore (e.g., 6,9–11]) neighbourhoods (four or eight neighbours, respectively), often with some directional component [12–14]. Some studies note that the geometry of the virtual landscape has the potential to affect simulation results [15,16], and the interaction strengths of orthogonal and diagonal neighbours in rasters are sometimes weighted using an appropriate algorithm [16,17]. Landscape permeability is sometimes defined using raster cells (e.g., [18]), although some authors have suggested using multiscaled rasters to represent patchy landscapes (e.g., [19]). Other studies use hexagonal geometry for both spatial analysis [20,21] and modelling [15,22–25] because the strengths of all neighbourhood interactions are equal. Irregular (variable shape and size) geometry has been used extensively in population modelling, but usually to parameterize or display discrete, spatially disparate habitat patches with explicit connectivity based on the distance between patches [26–28] or vector-based movement rules [29–31], and studies such as that by Ovaskainen [32] exemplify this approach. A few models use tessellated irregular shapes across a whole landscape, and implement cell-to-cell movement as part of the simulation [33,34], whereas Dunn and Majer [35] suggest that Voronoi (Dirichlet) cells are a convenient way to represent multiply scaled data, but they do not go into detail about dispersal mechanisms. However, no attempt has been made to specifically test how geometries other than rasters may affect movement in a mosaic landscape, and an explicit consideration of geometry does not appear to be an integral part of most population modelling studies.

The representation of real-world landscapes is complex, with the description of features represented as discrete objects subject to both qualitative and quantitative variability, uncertainty, or both [36]. Uncertainty within virtual landscapes is already considered in some disciplines (e.g., [37,38]). We suggest that all spatially explicit population models should consider how uncertainty in landscape representation may affect model output, just as sensitivity analyses on process model parameters have become standard practice. Clearly this is entirely dependent on the nature of the study undertaken (data, scale, structure, and discipline), so we cannot begin to describe how individual studies in diverse disciplines should address this issue. However, it seems inevitable that population modellers will adopt a probabilistic approach to spatial studies (which can be easily implemented through the use of alternative landscapes in successive runs of the model), and we provide a mechanism by which minimally biased landscapes can be created.

We created landscape mosaics with raster, hexagonal, and irregular geometries with which to model and compare the cell-to-cell exchange of information. We are not aware of any other study that directly compares the potential for systematic bias in the movement of information across the spectrum of possible geometries of mosaic virtual landscapes. We highlight how the geometry of cells in a raster virtual landscape affects both qualitative and quantitative aspects of spatial representation of irregular shapes, but leave the attributional representation of features (e.g., heterogeneous habitat [39,40]) and subsequent impacts on movement or process [41,42] to another study. We believe that these concepts are generally applicable across a broad range of spatially explicit modelling disciplines.

## Results

**Figure 1:**
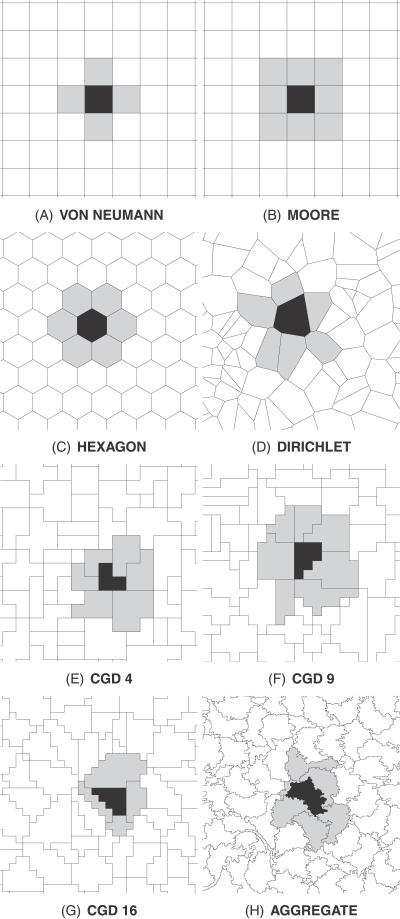
Example Instances of Eight Virtual Landcapes Example virtual landscape geometries (7 km × 7 km section). (A) von Neumann and (B) Moore neighbourhoods in a raster grid; (C) hexagonal; (D) Dirichlet tessellation; CGD tessellation with a mean of (E) four, (F) nine, and (G) 16 raster cells per km^2^; (H) land cover aggregate map. The neighbourhood (grey) of a focal cell (black) is highlighted in each virtual landscape.

**Figure 2:**
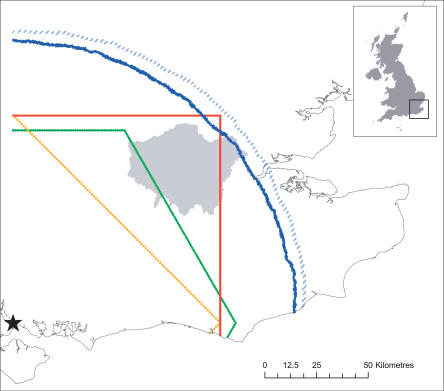
Maximum Distance Accessible in 100 Steps Maximum distance accessible in 100 cell-to-cell steps from the origin (star) in five virtual landscapes. The geometry of the regular grids is immediately apparent from the accessible regions of the von Neumann (yellow), Moore (red), and hexagonal (green) landscape models. Accessibility in all the irregular geometries was similar, and lay between that of the Dirichlet (circular blue line) and CGD4 (dashed circular blue line) virtual landscapes. London (shaded grey) is not accessible with some geometries, but is completely within reach of others.

We created eight virtual landscapes for comparison. Three virtual landscapes used a regular geometry: two rasters with von Neumann (Figure 1A) and Moore (Figure 1B) neighbourhoods and one of equilateral hexagons (Figure 1C). Virtual landscapes with irregular geometry were created in five different ways. One was a simple tessellation around random points (hereafter, the Dirichlet landscape; Figure 1D). Three virtual landscapes were approximations to the Dirichlet landscape, but based on a raster grid, with irregular cells composed of a mean of four, nine, or 16 squares (Figure 1E, 1F, and 1G, respectively) and called the coarse-grain Dirichlet (CGD) landscapes. The final irregular virtual landscape, called the aggregate map, was derived by aggregating habitat patches from real-world coverage data [3] and thus reflected the complex structure of a real landscape (Figure 1H). Ten instances of each irregular virtual landscape were created because all were formed with random processes. Only one instance of each regular virtual landscape was used. All landscapes were based in a real-world context (Figure 2).

### Characterizing Statistics

Cells in the raster and hexagonal virtual landscapes had a fixed number of neighbours (Table 1). Cells in the Dirichlet landscape had a mean of exactly six neighbours, though there was variation about this value within individual landscape instances. The CGD virtual landscapes all resembled pixelated versions of the Dirichlet landscape. However, both the visual and mathematical approximation improved as the resolution of the underlying raster was increased, as demonstrated by both the mean and standard deviation of the number of neighbours (Table 1). Cells in the aggregate map had approximately six neighbours, and were a range of shapes because the sequential building rules meant that growing cells were often geometrically constrained by neighbours. Of the geometries tested in this study, the mean number of neighbours of a cell was six, or its approximation, with the exception of the rasters. There was variation in the distribution of cell sizes within the irregular virtual landscapes (Table 1).

**Table 1:**
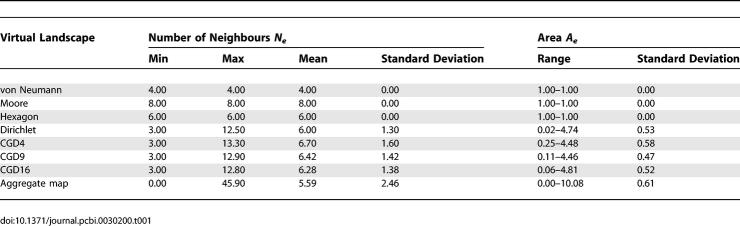
Description of Area and Neighbourhood for Parcels in the Interior of All the Virtual Landscapes

**Figure 3:**
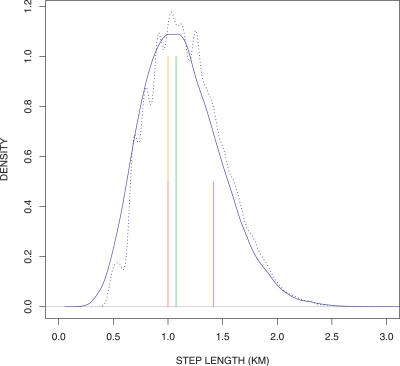
Distribution of Step Lengths The distribution of step lengths possible in five virtual landscapes. The von Neumann (orange, at 1.0) and hexagonal (green, at 1.074 km) landscapes only allow a single step length, whereas the Moore geometry allows two steps (red, at 1.0 and 1.41). A single instance of a Dirichlet landscape (blue, mean 1.095 km, gamma distributed with shape = 1.98, rate = 1.8 × 10^4^) allows a distribution of step lengths that vary from parcel to parcel but have a mean similar to the hexagonal geometry. Other irregular landscapes have step length distributed similarly (CGD4 shown, blue dashed line, mean 1.15 km).

We measured and compared all possible unique cell-to-cell step lengths (measured between centre-of-mass centroids) in five landscapes: the three regular landscapes, and single instances of the Dirichlet and the CGD4 landscapes (Figure 3). In the von Neumann and hexagonal landscapes, only one step length was ever possible, with lengths 1 km and 1.074 km, respectively. In the Moore landscape, two steps were equally probable, with lengths 1 km and 1.41 km producing a mean step of 1.21 km per landscape. Step lengths in the Dirichlet landscape were gamma distributed (Figure 3) with a mean of 1.095 km, which is close to that found in the hexagonal landscape; the step lengths of each cell in the CDG4 landscape were similarly distributed with a mean of 1.18 km, though the distribution was less smooth as a result of the finite distribution of cell shapes and hence step lengths (Figure 3).

### Moving across Model Landscapes

#### Accessibility.

We used three methods to investigate movement (of individuals or information) across our virtual landscapes; these were accessibility, random movement, and directed random movement. Accessibility (sensu [43]) measured the shortest possible sequence of cell-to-cell steps between two points in the virtual landscape. We implemented this as the maximum geographical distance accessible from a common origin in a fixed number of steps (Figure 2). There were striking differences between the accessibility of the regular virtual landscapes and those with an irregular structure (Figure 2). The mean minimum steps required to access a fixed distance (effectively the inverse of Figure 2) varied considerably between the regular models (to travel 100 km took a mean of 125.9, 90.7, and 102.6 steps for the von Neumann, Moore, and hexagonal virtual landscapes, respectively) and were large compared to the mean minimum steps required in the irregular landscapes (approximately 73 steps in all five irregular landscapes).

**Figure 4:**
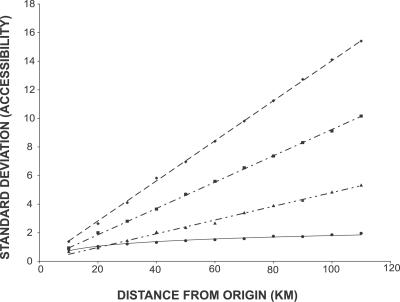
Angular Variation in Accessibility The minimum number of steps required to travel a range of distances was measured every 10° across a 90° angle in four virtual landscapes. The standard deviation increased linearly with increased distance (*d*) in the regular grids; von Neumann (dashed line) has trend 0.14*d* (*R* ^2^ = 0.9995), Moore (dash dot) 0.09*d* (*R* ^2^ = 0.9994), and hexagons (dash dot dot) 0.05*d* (*R* ^2^ = 0.9952). Standard deviation in the Dirichlet virtual landscape (solid line) increased with trend 0.46 ln(*d*) (*R* ^2^ = 0.968).

There was considerable directional bias shown in the accessibility of the three regular virtual landscapes. The maximum distance accessible in a fixed number of steps in the von Neumann, Moore, and hexagonal landscapes produced a distinctive shape dependent on their neighbourhood rules: a diamond, a square, and a hexagon, respectively. In contrast, accessibility in the irregular virtual landscapes was always circular. The angular variation in maximum distance accessible is demonstrated numerically by the standard deviation of the minimum number of steps required to travel a fixed distance in any direction (Figure 4). The degree to which this represents a problem is dependent on the resolution and extent of the virtual landscape (i.e., size measured in cells). The standard deviation of the minimum number of steps needed to travel a distance *d* increased linearly with an increase in *d* in all three regular geometries (Figure 4). However, standard deviation in the irregular geometries only grew with log(*d*), and thus effectively asymptotes in most real-world applications. Virtual landscapes built with irregular geometry appear unequivocally superior to those built with regular geometry, because they have no directional biases in accessibility.

There is an apparent connection between the circle described by the irregular landscapes and the maximum distance possible in the Moore landscape (i.e., the northeast corner of accessible cells in Figure 2). We measured the maximum step length possible from each cell in the Dirichlet landscape. These had a mean of 1.47 km, which is similar to (if slightly larger than) the longest possible step in the Moore landscape, which is diagonal (1.41 km). By definition, the accessibility metric connects two distant cells by the smallest number of steps possible and is therefore proportional to the mean maximum step length. This is the basis of the coincidence between the circle describing accessibility in the Dirichlet landscape and the diagonal movement described in the Moore landscape.

#### Forced and unforced random movement.

**Figure 5:**
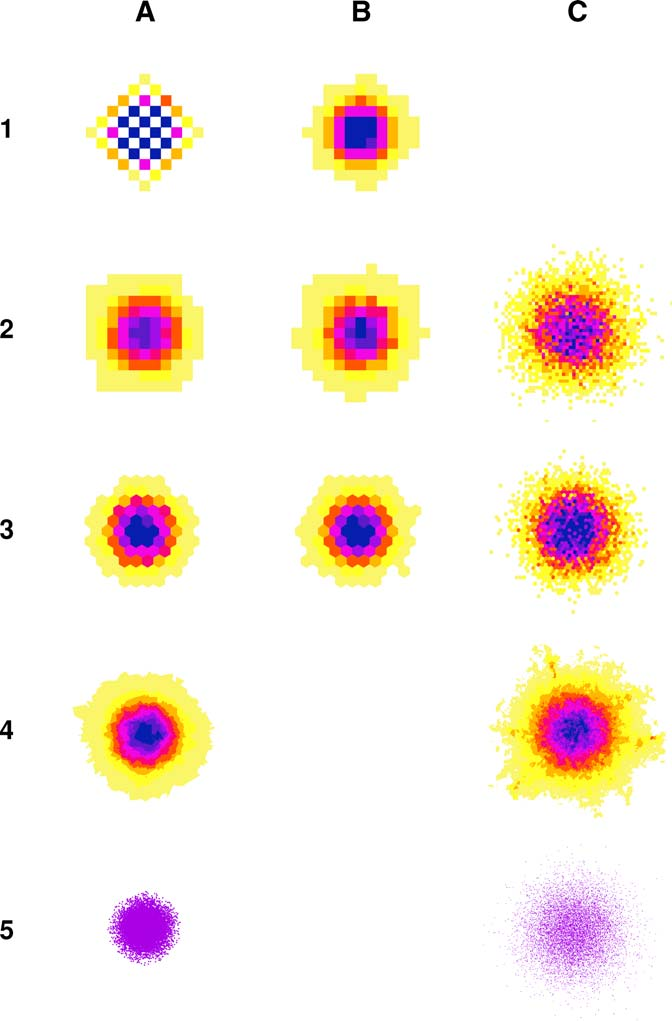
Population Distributions after Random Movement in Different Virtual Landscapes A matrix showing population distributions after a number of random movement scenarios. Rows (from top to bottom): (1) von Neumann; (2) Moore; (3) hexagon; (4) Dirichlet; and (5) vector landscapes. Columns (from left to right): (A) random movement with *t* = 5 (time steps) and *p* = 1 (probability of movement in a time step); (B) random movement, *t* = 10, *p* = 0.5; and (C) random movement, *t* = 100, *p* = 1. Colours represent population density in each cell on a common scale, ranging from yellow (low density) through orange, red, and purple to blue (high density). In row 5, the vector points are only represented in one colour.

We investigated the spatial distribution of a single population after completely random movement of its constituent individuals between neighbouring cells (i.e., uniform probability of movement to any neighbour). Three variants were tested in which movement was forced for all individuals at every step in short walks (Figure 5, column A) and in long walks (Figure 5, column C), and where only a proportion of individuals moved in each time step (Figure 5, column B). We made a simple statistical comparison with the population distribution after a vector random walk at an equivalent scale (number of individuals, time, and probability of movement) as a benchmark (Figure 5, row 5).

The regular virtual landscapes resulted in obviously structured population distributions after a small number of steps. The von Neumann grid was the worst, with every alternate cell being unoccupied because all individuals were forced to move in every time step (Figure 5, A1). This pattern was independent of the length of the movement. The Moore and hexagonal landscapes also showed strong influences from their underlying geometry (Figure 5A2 and 5A3). Even when only a proportion of the population moved in each time step, a strong pattern reflecting the regular geometry of the virtual landscape was still evident (Figure 5B1-5B3); whereas the Moore and hexagonal landscapes compared favourably with the benchmark along the north–south cross-section (Pearson correlation coefficient, *N* = 9, *r* = 0.997 for both comparisons) at other angles, and for any cross-section of the von Neumann landscape, population distribution differed significantly from the benchmark (Pearson correlation coefficient, *r* between 0.5–0.6 for all comparisons). Increasing the length of the random walk produced more visual circularity in the population distribution in the Moore and hexagonal landscapes (Figure 5C2 and 5C3). The irregular virtual landscapes all showed circular population spread, regardless of the scale of the random walk (Dirichlet landscape shown in Figure 5A4 and 5C4; for clarity and because of the extreme similarity, other irregular virtual landscapes are shown in Figure S1). The number of individuals at distance *d* from the centre was the same as the benchmark (Pearson correlation coefficient, *r* > 0.98 for all paired comparisons), and no geometric structure was evident under any of the irregular virtual landscapes. Longer random walks showed less geometrical bias, and the distribution of the population was statistically indistinguishable between all virtual landscapes (Figure 5, column C), and the same as the relevant benchmark (Pearson correlation coefficient, *r* > 0.9 for all paired comparisons). In summary, all geometries, with the exception of von Neumann, showed no directional bias for long random walks. However, regular virtual landscapes showed characteristic biases for shorter walks, whereas the irregular virtual landscapes performed uniformly well across all scales.

#### Forced and unforced directed movement.

We investigated the spatial distribution of a single population after directed movement of its constituent individuals, which replicates a dispersal event more similar to animal invasion and disease models in which movement is sensitive to its history of progress. Movement was directed away from the cell containing the origin by preferentially choosing neighbours no more accessible from the origin, with both forced (all individuals moving at every time step) and unforced walks. Three approaches were used: forced movement with no backward component, unforced (50% chance of movement) with no backward component, and forced with 10% backward movement.

**Figure 6:**
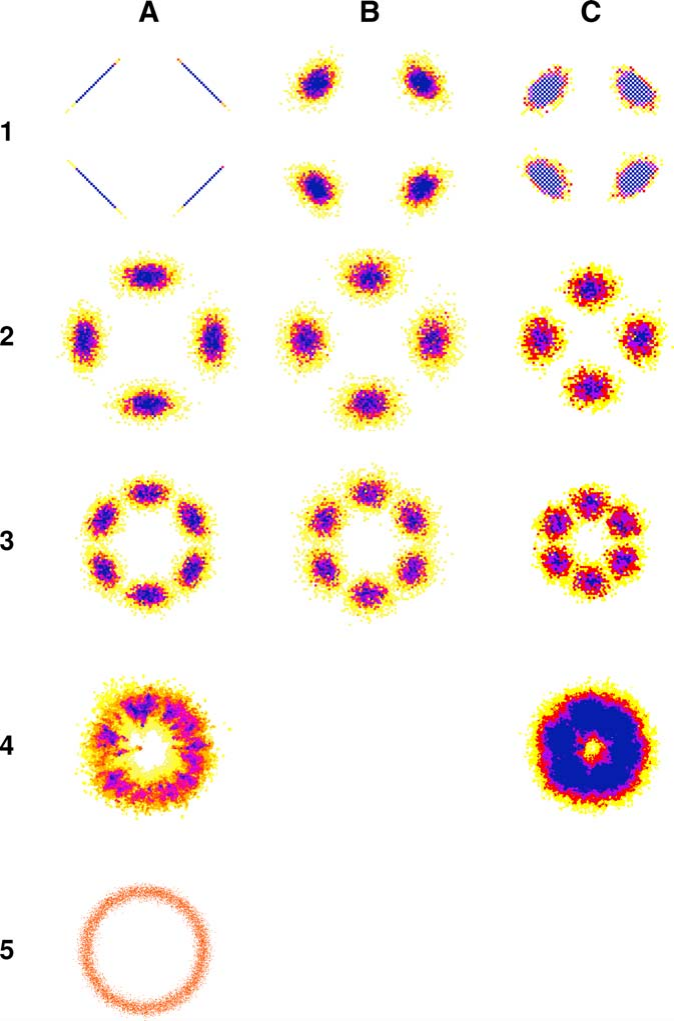
Population Distributions after Directed Random Movement in Different Virtual Landscapes A matrix showing population distributions after a number of directed random movement scenarios. Rows (from top to bottom): (1) von Neumann; (2) Moore; (3) hexagon; (4) Dirichlet; and (5) vector landscapes. Columns (from left to right): (A) directed random movement, *t* = 50, *p* = 1, *b* = 0.0 (probability of backward movement); (B) directed random movement, *t* = 100, *p* = 0.5, *b* = 0.0; and (C) directed random movement, *t* = 50, *p* = 0.5, *b* = 0.1. Colours represent population density in each cell on a common scale, ranging from yellow (low density) through orange, red, and purple to blue (high density). In row (5), the vector points are only represented in one colour.

In a raster or hexagonal virtual landscape, forced movement with no option to move backward resulted in population distributions with clear, regular geometric patterns (Figure 6). Individuals aggregated into subpopulations located midway between the vertices accessible by maximum possible displacement (Figure 6A1-6A3); thus the raster geometries had four subpopulations, whereas the hexagonal geometry had six. Individuals in the von Neumann grid (Figure 6A1) all reached maximum displacement because no cells had neighbours at the same distance from the origin, making radial movement impossible. Even after unforced movement with no backward component, regular landscape geometry significantly affected population distribution (Figure 6B1-6B3), although the von Neumann grid was less extreme under this rule. Forced movement with a backward component still exhibited clustering in the regular landscapes (Figure 6C1-6C3). Unpublished data suggest that the strength of the clustering of the population in regular landscapes is proportional to the length of the random walk, and thus we anticipate greater biases will occur with long walks across large, regular landscape extents.

All directed movement across any irregular landscape resulted in an annular (ring-shaped) population distribution (Figure 6A4 shows the Dirichlet landscape; other irregular landscapes are shown in Figure S1) with no visible geometrical influence and similar to that seen in the benchmark vector walk (Figure 6A5). Although not demonstrated in the figures, the geometric form of the population distribution across irregular geometries was not a function of the length of the walk. In summary, the regular geometries showed strong directional biases with short walks, and the biases got worse as the number of steps increased. Conversely, the irregular geometries showed repeatable and scale-independent behaviour that largely approximated idealized vector random movement for all but very long walks.

We note that the net displacement of all populations in discrete virtual landscapes was smaller than found in the continuous benchmark landscape. In a directed vector random walk across a continuous landscape, all steps had a positive outward component, and the effect became more marked with increased distance from the origin. However, in the discretized landscapes, net displacement was reduced because individuals were able to move circumferentially or into cells whose centre of mass was geographically closer to the origin.

#### Movement proportional to the shared boundary.

It might be argued that previous movement rules, with uniform probability of neighbour choice, are overly simple. To test this, we reran three of the previous walks in a single Dirichlet landscape, with the probability of movement into neighbouring cells proportional to the length of the shared boundary. Movement into neighbours that shared long boundaries were favoured over those that shared short boundaries. The von Neumann and hexagonal landscapes consist of equilateral shapes, so neighbourhood choice rules based on proportional boundary length resulted in uniform choice, as tested previously. We did not test the Moore landscape because it reduces to the von Neumann landscape under this rule. The three walks tested were a forced random walk, a forced directed walk with no backward component, and a forced directed walk with some backward component.

**Figure 7:**
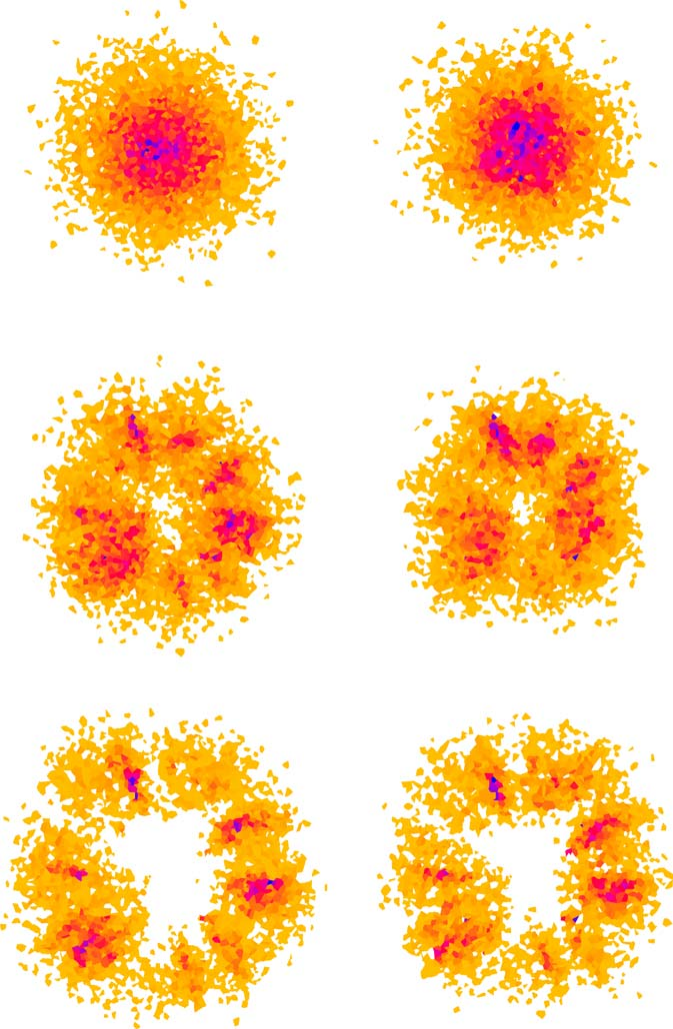
Random Movement in a Single Dirichlet Landscape Population distributions in a Dirichlet landscape are shown after: (top) random movement with *t* = 100 (time steps), *p* = 1 (probability of movement in a time step); (centre) semi-directed movement with *t* = 50, *p* = 1, *b* = 0.1 (probability of choosing a neighbour closer to the origin); and (bottom) directed movement with *t* = 50, *p* = 1, *b* = 0. In the left column, individuals move into a neighbouring parcel with probability 1/(number of neighbours). In the right column, individuals move into a neighbouring parcel with probability proportional to the length of the shared boundary. There is no significant difference in the population distributions despite the difference in neighbour choice.

Changing a uniform choice of neighbours to a choice proportional to the length of the shared boundary did not qualitatively change the distribution of the population for any of the movements tested (Figure 7). The length of the shared boundary is just one geometric function that could be used to weight movement into neighbouring cells (others include proportional area of the neighbours, distance to the centroids, etc.). In an irregular landscape in which these attributes are distributed randomly, we suggest that none of these will produce significant differences from the uniform choice, and hence will not introduce bias in the movement of the population across the mosaic landscape.

### Representing Real-World Landscapes Using Iterated Rasters

We measured the land area from a number of raster depictions of a fine-scale vector description of a real-world object (United Kingdom (UK) coastline). Rasters were created at a range of scales with a variable origin (shifted successively by 10% of the resolution west and south). A further ten rasters, at a resolution of 10 km, were created with a fixed origin but with the orientation of the grid rotated successively by 9°.

**Figure 8:**
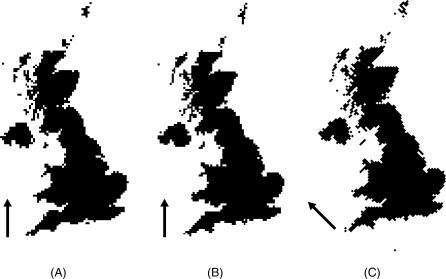
Qualitative Differences in Raster Representation of a Real Extent Three alternative raster representations of the UK at 10 km resolution, formed against the BNG. In (A), the origin is at BNG (0,0) and the raster is aligned with the BNG. In (B), the origin has been shifted to BNG (−5000,−5000) but the orientation is unchanged. In (C), the origin is at BNG (0,0), but the raster has been rotated by 45°. The arrows refer to the orientation of the raster grid. Observe the variation in shape and size of the Orkney and Shetland isles (the two groups of islands north of the mainland).

**Figure 9:**
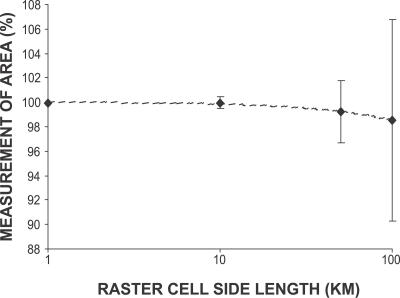
Variation in the Area Measurement of a Real Extent The area of the UK was measured from raster datasets at resolution 1, 10, 50, and 100 km, as a percentage of a vector polygon area (245,660 km*^2^*), and the mean calculated. The *y*-error bars denote coefficient of variation. The mean is always an underestimate, and worsens at lower resolutions.

The qualitative form of a raster representation of the UK differed with a change in origin or orientation of the grid (Figure 8). Small objects such as islands appeared or disappeared, became connected or disconnected from the mainland or each other, or changed their shape radically. This would have clear effects on any model involving terrestrial movement. Although we have only shown this at one scale, these undesirable effects are fractal, and would be present as a possible bias at all scales, and would worsen at larger resolutions [44]. The mean area reported by the raster representations of the UK decreased with increasing scale (Figure 9), though as a fractal property, would show bias at all scales. The best mean estimate (99.9% of the vector original) was derived from the finest scale raster (1 km), but mean estimate of national area fell to as low as 98.5% at a 100 km resolution. In addition, the variance around these mean figures was considerable and also increased with scale. The worst performing rasters (two out of ten instances at a 100 km resolution) showed an area of only 89.6%, an alarming loss of 10.4% of the British land surface. The estimates of national area produced by iteration across multiple rotations of a raster grid at 10 km resolution had a mean of 100%; individual instances ranged from 99.3% to 100.6% of the vector original, suggesting that at this scale, the orientation of the raster had only a small quantitative effect on area.

All forms of raster representation have biases, variant with scale. In contrast, an irregular geometry (specifically a vector representation) can be subdivided into as many randomized vector cells as necessary. Any estimate of gross area, length, or geographic property is perfect (zero bias). When multiple instances of virtual landscapes are required, we recommend the use of irregular geometry to avoid introducing bias to representation of its extent.

## Discussion

The reduction of bias in model output should always be a priority (e.g., [45,46]). Biases can be hidden but still present in many components of a model, and their presence or interaction may produce artefacts. Sources of bias should be looked for, and where present, measured in order to decide whether they are significant in the context of the results. If they are significant, the bias should be minimized or, preferably, removed altogether. Movement in spatial models is such a fundamental process that bias in this process is likely to be critical. Some models are entirely theoretical and are used to gain insight into academic problems, in which some forms of bias may be acceptable. However, our focus is primarily on predictive models designed to support decisionmaking, where high confidence is required in the results and validation is difficult. In these models, it is essential that sources of potential bias be removed, but we believe that it is desirable in all types of models if the interpretation of results may be confounded.

We show that the geometry used to implement cell-to-cell movement has the potential to bias a diverse array of movement rules for information flow across landscapes. We tested completely random movement at one extreme, completely directed movement at the other extreme (accessibility), and a number of directed random movement rules with both forward and backward components. Other choices in this continuum were possible; however, we did not wish to test the complete library of walks ever created, but merely to demonstrate the potential for bias across the spectrum of movement types. To this end, all of the movement rules were short and simple, and the virtual landscapes homogeneous, in order to test the geometric properties of the virtual landscape and not those of the movement. We are aware that some of these methods may appear unrealistic or extreme to some disciplines. Less extreme movement rules may produce less biased results, but we would argue that some bias still exists. Inevitably the details of how cell-to-cell movement is expressed will vary between disciplines and studies, and will also be affected by scale, so we make no attempt to advise on the use or abandonment of any method of movement. However, we do wish to highlight the fundamental interaction between landscape geometry and movement, and present the use of irregular landscapes as a potential solution to some of the biases that may be encountered.

### Movement across Landscapes

Simple random walks in virtual landscapes with regular geometries can produce enormous qualitative biases in the direction and extent of movement and hence bias the distribution of populations in space. For all our investigations (accessibility, random movement, and directed random movement), the regular geometries performed poorly at some or all scales for measures of both distance and direction. The nature and strength of the bias was, in part, a function of the length of the movement and the resolution of the landscape, with the potential for different biases to worsen after both short-distance and long-distance movement. In an ideal homogeneous virtual landscape, the distance travelled by an individual (from its origin) after random movement should be independent of the direction travelled at each step. Regular grids all restrict the direction of movement to the same few angles at every step, so the final positions of individuals cannot be independent of the grid structure. Even if the scope of the movement neighbourhood is extended to include more distant cells (e.g., to include the 16 cells adjacent to the eight immediate neighbours in the Moore neighbourhood), so that single steps may include jumps over the immediate neighbours, a regular geometry restricts the available directions to some degree. In comparison, the direction of neighbouring cells in a single, irregular virtual landscape is not set by the geometry, and when enough multiple irregular virtual landscapes are considered together, available directions assume a uniform circular distribution.

**Figure 10:**
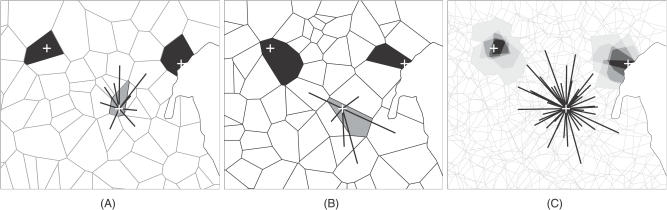
Space and Direction from a Fixed Point in Multiple Irregular Landscapes (A) A single Dirichlet landscape showing three fixed points (+). One (top left) occupies a cell inland, while another (top right) occupies a coastal cell that is restricted by the edge of the extent. The third (centre) is shown with the available directions for movement in that landscape instance. (B) A second random instance of a Dirichlet tessellation in the same extent. The three fixed points are highlighted, with their respective cells and directions of movement. (C) The sum of the observations of space and direction around the three points after only five Dirichlet landscapes. Light grey lines indicate the boundaries of cells in all five landscapes. The kernel associated with inland and coastal points is shown in shades of grey, with the lightest shade showing an area only associated with the point in one landscape instance and black being the area common to all five. Although clearly not circular, given enough iterations, all points approach a circular kernel of influence unless restricted by the extent. Available directions for movement across all five landscapes are shown from the third point, demonstrating that movement in any direction is equally possible.

We demonstrated that, in the same way that regular landscapes restrict the available directions for movement, they severely restrict cell-to-cell step lengths, and in part, this may explain a component of the bias in movement. The hexagonal landscape is the optimum arrangement of circles packed in space using a single iteration. Dirichlet landscapes emulate circular cells over many iterations (Figure 10), and the mean step length closely approximates that of the hexagonal landscape, even in a single iteration. Although the step lengths in the CGD landscapes followed the same general distribution as the vector Dirichlet landscape, individual step lengths were more or less frequent than expected. We suggest that although the results of random walks appeared similar in all the irregular landscapes, a smooth distribution of step lengths is less likely to be a source of bias, and therefore, at fine scales in particular, we recommend the vector Dirichlet landscape over the CGD irregular landscapes.

We propose that the average number of neighbours per cell is a useful metric for quantifying the potential for landscape geometry to introduce bias in movement. A mean close to six neighbours appears to be the ideal; all the irregular landscapes have this property. Although the hexagonal landscape has six neighbours per cell, the variance is zero, and therefore directional movement must be restricted; we therefore extend the metric to include a nonzero variance. The square geometries fail in both respects.

Because we have identified the potential for bias in movement in regular geometries, we believe that studies need to show (not assume) that movement in their own spatial models produces no bias. Bias due to geometry may not be apparent in models with complex rules for cell to adjacent cell movement, e.g., dependence on heterogeneous landscape quality (e.g., habitat preference [6] and permeability [47]). Where sources of geometrical bias have been identified, individual studies have attempted to compensate with a variety of approaches [16,17]. Not only is this compensation dependent on the spatial and temporal scale of the model for which it is designed, it adds more potential for bias to the model (albeit pulling in the opposite direction) and may adversely interact with other components of the model. There is no way to be certain that biases masked at one scale will not produce artefacts when a predictive model is extrapolated in time or space. In contrast, there is no potential for bias in both the direction and distance of movement of individuals across virtual landscapes with irregular geometry.

### Representation of Whole Landscapes

Some of the deficiencies of cell-to-cell movement across regular landscape geometries identified here might be overcome by iteration of rasters, specifically by randomizing origin and orientation. However, this process can change the quality of the representation of the whole landscape, and it is this that we concentrate on here. The dependence on scale in the adequate representation of complex shapes with rasters has been well-discussed elsewhere [44,48–52]. Our representation of the UK coastline, and the measurement of its area, although not novel, allowed us to focus specifically on the qualitative and quantitative effects of using multiple regular geometries to represent a complete extent. We used the UK landmass as an example of a real-world object that has an irregular extent whose boundaries could not be defensibly redefined as a regular shape for a national-scale model. The representation of real-world features with regular geometries must always be an approximation [50] whose adequacy can only be measured by model output. We have shown that at any finite scale, a feature represented by a single virtual landscape with regular geometry will always show qualitative and quantitative errors. Some of these errors can be extreme, and the modeller choosing a single instance of a regular geometry with which to represent the landscape has no way of knowing how adequate it is without testing several representations. Biases in the mean output could be reduced through iteration of regular geometries (with random origin and orientation) and a careful choice of scale, but the interpretation of results would only be acceptable where the bias was quantified. In contrast, any landscape subject to a spatial modelling study can be split up into irregularly shaped cells with no detrimental qualitative or quantitative effect on the representation of the whole. If accuracy in feature representation is important, the superior alternative to the raster is the irregular and vector-based mosaic.

### Scale

The problems of feature representation by regular geometries are compounded by the connection between scale and structure; the form and significance of any feature alters as soon as the scale is changed. This is especially problematic where interacting processes occur at different scales in the same landscape. Because scale has such a strong effect on the properties of model output, it is a pity that studies suggesting quantitative methods for determining the most appropriate scale have not been pursued (but see [53] for a generic approach, [54,55] for more specific applications). In the absence of quantitative rules, modellers have to rely on common sense or experience to choose scale, and therefore should clearly demonstrate that their choice is appropriate by showing that the bias produced by the model at that scale is acceptable.

The choice of resolution in many raster-based models to date is often either derived from technical data (e.g., satellite imagery) (e.g., [39,56]) or chosen to be the nearest integer measurement of an apparently relevant process (usually biological; e.g., [4,6,57]). We have shown that the potential for bias is always present in regular virtual landscapes even at high resolutions, and the impact of that bias is a function of scale. If only one raster landscape is used in a model, its origin and orientation appear arbitrary yet are usually unchallenged (this is apparent from the lack of ability to rotate rasters away from a north–south orientation in some GIS packages). If the resolution of the raster is high enough, the representation of features will be little changed by origin and orientation, but model processes may be affected. However, if the data or the process suggests modelling at a low resolution, using a raster must sorely compromise the representation of the landscape.

Using irregular mosaic landscapes solves two problems. First, the interaction of bias with scale is removed from the process model (c.f., directional bias in movement in this study). Second, the quality of the spatial representation of available data no longer depends solely on resolution; landscapes formed from vector-based, irregular cells can remain faithful to the available data at any scale and in any single instance. The modeller is thus free to set a scale appropriate to other model processes.

### Landscapes as Models

Lindenmayer, Fischer, and Hobbs [1] emphasize that the ability to choose one of a number of landscape models is important in fauna research. We suggest that, in the mosaic-based landscape paradigm, the automatic use of multiple landscape models should be widespread. Real landscapes comprise irregular and complex shapes [58] that can only be well-described using irregular cells. There will always be quantitative uncertainty in the location and boundary shape of cells and qualitative errors in their internal description even if irregular virtual landscapes are created deterministically from underlying habitat patch data rather than randomly (e.g., Dirichlet polygons). The only way to explore and incorporate the uncertainty associated with landscape representation is to model with multiple, alternative virtual landscapes and present results as probabilistic maps (e.g., [59]). This applies equally to regular and irregular mosaic landscapes.

The computational demands of running irregular models such as the ones in this study are not necessarily more than those of a raster model. Because the movement rules are so simple, all that is required to implement movement is a list of cell IDs and associated neighbours. All the investigations in this study were run in Python, only using the GIS for creating and displaying landscapes. We admit that preparation of an irregular landscape set requires some extra work; however, if it is accepted that multiple virtual landscapes are necessary (e.g., different random centroids for Dirichlet tessellations, and different origins and orientations for rasters), the effort in preparing irregular and regular landscapes becomes almost indistinguishable. The irregular landscapes also appear to yield greater returns, since they are scale-independent and bias-free, and can represent features within the landscape as well as the available data allow. It is worth pointing out that the ideal vector implementation of an irregular mosaic landscape can be approximated very easily by a raster aggregate; the use of models such as the CGD (Figure 1E–1G) brings the benefits of unbiased movement, although the best representation of edges and features is only achieved by using a very high resolution raster.

We have shown that all single instances of irregular mosaics have similar structure (number of neighbours) and statistical properties (e.g., step length distribution), which results in scale-free and similarly unbiased movement of information (populations). However, the structure and statistical properties of regular mosaics differ from each other and from that of the irregular mosaics, resulting in biased movement that cannot be easily compared. We suggest that two spatial models using irregular virtual landscapes of any scale may be more easily compared than those using regular geometry, which must have both the same scale and the same structure, and therefore the same bias.

### Further Benefits

We believe that the use of irregular geometry to form virtual landscapes may bring many additional benefits. We illustrate these with examples from population modelling, in which information has an integer form (e.g., individuals), but the principles should be applicable in many disciplines, including those concerned with the movement of infinitely divisible information (e.g., water). The area and shape of cells in an irregular geometry can vary across the extent of the virtual landscape so that regions requiring a detailed spatial description (complex habitat patches, linear features such as rivers, etc.) can be represented either with a greater density of irregular cells or exclusively with a single cell. This property of irregular landscapes has also been identified by Dunn and Majer [35] and is analogous to the raster approach of Tischendorf [19]. In turn, this permits an improvement in the description of cell attributes and reduces their uncertainty. This is especially important where cell attributes have the ability to affect the spatial output of the model (e.g., landscape connectivity, patch permeability, and population persistence [60]). We have validated how the aggregation of real-world features into irregular cells can provide a sufficiently irregular virtual landscape to avoid bias (i.e., aggregate map, Figure 1H).

Another useful benefit of irregular geometry is illustrated by considering a fixed point in space. A single irregular cell containing this point is obviously not circular, but cells taken from sufficient iterations of the virtual landscape will approximate a circular kernel around the point (Figure 10). This concept is useful both in describing individual behaviour (i.e., zones of perception) or group dynamics (i.e., social interaction and density dependence). Most dispersal kernels in continuous space implicitly define movement to nearest neighbours as the most frequent, with vector movement resulting in individuals moving preferentially to sites that are close [61]. By using irregular cells, such kernels can be tuned with biological and geographical realism (e.g., interaction groups are bounded by major roads or coastline, perception zones do not include impenetrable habitat, and movement cannot cross rivers; see Figure 10). Finally, we observe that the geometry and size of real-world processes and objects (such as home ranges, social group territories, habitat patches, and habitat quality) are irregular and variable ([58]; specific examples include the spatial arrangement of subpopulations of rabbits [62], badgers [63], and coyotes [64]). There is a significant body of literature in identifying habitat patches in real-world landscapes (e.g., [65–67]). Their subsequent representation with single cells of regular geometries is inappropriate. In addition, if there are few data on how cells are formed in the real landscape, the most defensible way of expressing them in a model is through multiple, randomly generated (irregular) mosaic landscapes.

### Conclusion

This study was prompted by a desire to construct a universal framework within which uncertainties in landscape description could be included explicitly in model function and results, and in which movement could be modelled as simply as possible in an unbiased, generic, and flexible manner.

A single virtual landscape formed with regular geometry has a huge potential for bias. In contrast, this study has shown that even a single virtual landscape formed with irregular geometry has no potential to bias the direction or distance of movement of information (e.g., individuals), and although the defensible use of a random property (in this case, geometry) requires multiple instances, the variation between irregular landscapes is small.

Any representation using a regular geometry is at best a good approximation. Even when multiple instances of regular geometries with random origins and rotations are measured, the mean output still has the potential for bias, and the variation between instances is large. The representation of an extent by an irregular geometry shows no error.

We recommend the use of irregular geometry and multiple random instances in creating any virtual landscape, which eliminates bias in the movement of information and the representation of real-world extents. Both are specifically recommended for models that are designed to help make decisions, so that the probabilistic output encompasses the uncertainty in both population processes and spatial representation. As this is the first study recommending the use of irregular geometries, and we have not covered issues of internal representation (e.g., small features and heterogeneous landscape quality), it is difficult to state unequivocally that they are completely superior to regular geometries, but the results presented here suggest that they should be the first choice for modelling virtual landscapes.

## Methods

### Creating and characterizing model landscapes.

**Figure 11:**
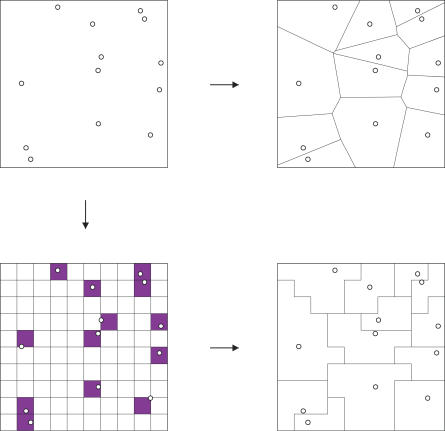
Creation of a Coarse-Grain Dirichlet Landscape from a Raster To create a Dirichlet landscape, starting points are chosen randomly (top left). The vector Dirichlet landscape (top right) is shown for comparison. Random starting points are translated into a raster grid (bottom left). All other raster squares are then assigned to the nearest coloured square (measured between centroids) and boundaries dissolved to produce the CGD landscape (bottom right).

Our virtual landscapes were created to have a mean cell area of 1 km^2^ across the study area, which was a 25,361 km^2^ area of southeast England. The two rasters used an arbitrarily chosen origin of (0,0) on the British National Grid (BNG); the placement of the hexagonal landscape was entirely arbitrary. The Dirichlet landscape was formed by a vector tessellation using the ArcInfo Thiessen on 25,361 random points drawn from a uniform distribution within the study area. The coarse-grain landscapes were created from three raster landscapes (origin at 0,0 BNG), with 500-m, 333-m, and 250-m resolutions. Within each raster, all squares were associated with, and then aggregated to, the nearest of 25,361 randomly chosen squares (see Figure 11 and pseudocode at the end of this section). The aggregate map was created from Land Cover 2000 [3], a fine-scale vector habitat coverage. A random habitat patch was selected and neighbours absorbed in turn until a total area of 1 km^2^ ± 5% was achieved. The neighbour with the next-closest centroid was always chosen with the aim of maximizing circularity; other joining rules are possible, e.g., habitat similarity, but the geometric optimization was chosen for its simplicity.

The neighbourhood of any focal cell in the hexagonal and irregular landscapes was defined as all cells in the landscape with a boundary (or point on the boundary) shared with the focal cell. The interior of a virtual landscape was defined as all cells that were more than 1 km from its boundary. The number of neighbours (*N_e_*) and area (*A_e_*) of interior cells were measured across all virtual landscapes and all instances on irregular landscapes. The minimum, mean, maximum, and standard deviation of *N_e_* and *A_e_* were calculated for each virtual landscape.

### Moving across model landscapes.

Probes were developed to quantify the effects of landscape geometry on cell-to-cell movements, and are based on the following generalized approach. In a discrete landscape, individuals move from one cell *e* into any neighbouring cell with probability 1*/N_e_*, where *N_e_* is the number of neighbours of *e*. We compared the performance of the probes against the simplest vector random walk. This unbiased benchmark moved an individual a fixed distance (1 km) from its starting position (*x,y*) at a randomly chosen angle in (0,2π). Movement probes always consisted of 10,000 independent individuals. Irregular virtual landscapes used 1,000 individuals across ten randomized instances, with figures and statistics presented as the sum of the instances (see Figure 10).

For the accessibility probe, we calculated the minimum number of steps required for an individual in any cell in the landscape to access the origin (cell containing BNG 448500, 104500; southwest corner) in all virtual landscapes. We mapped the limit of the region accessible from the origin with 100 steps and measured the mean of the minimum number of steps required to reach a range of distances from the origin at 10° angles. The random movement probe started in a cell containing the origin (BNG 524500, 179500; centre of virtual landscape). Vector random walks started at the origin. We used four sets of parameters (probability of movement, *p*, number of time steps, *t*) to simulate a variety of forced and unforced, short and long walks: (*p =* 1, *t =* 5), (*p =* 1, *t =* 100), (*p =* 0.5, *t =* 10), and (*p =* 0.5, *t =* 200). Direct comparison of population displacements can only be made between probes where the product of *p* and *t* is equal, thus unforced walks last twice as long as equivalent forced walks. The population distribution also was calculated for directed random walks, in which neighbouring cells were divided into “backward” (toward the origin) and “forward” (around or away from the origin) neighbours. Fully directed (no backward movement) and semidirected (10% possibility of movement backward) random walks were simulated for (*p =* 1, *t =* 50) and (*p =* 0.5, *t =* 100). Directed movement was implemented in the vector random walk by disallowing any choice of angle that would result in a new location closer to the origin. Notice that in a totally random walk across a landscape with mean of six neighbours, backward movement would be achieved on average in two out of six cell-to-cell movements (with two radial movements and two forward movements also possible), or with 33% chance. Therefore, a 10% chance of backward movement in the directed random walk reduces backward movement by one-third and not by one-half (if movement was only forward or backward).

For statistical tests of similarity, population density (individuals per square kilometre) was measured in the cell containing locations 0,1,2,3,... km from the origin, at angles 0°, 60°, and 90° from north. The number of individuals within a circle with area 1 km^2^ centred at these locations after the equivalent vector movement was used as a benchmark. Pearson's product moment correlation coefficient was calculated for the paired datasets for each cross-section. For short random walks, locations from 0,..,8 km were used; for long walks, we used 0,..,36 km.

### Representing real-world landscapes using iterated rasters.

Using a vector polygon of the British coastline (of area 245,660 km*^2^*), we created ten raster representations of the UK using a resolution of 1, 10, 50, and 100 km. At each scale, the origin of the raster (lower left corner) was initially set at BNG 0,0 with nine further representations created by moving the origin 10% of the resolution, both west and south.

### Creation method for a coarse-grain Dirichlet landscape.

Pseudocode for creating a CGD landscape with mean cell size of 1 km^2^ is shown below. See Figure 11 for an illustration of the process.

Let D be an X by Y grid with cells d_x,y_:

x,y are the coordinates of the centroid of the raster cell

d_x,y_.belongs_to = null

d_x,y_.id = (X − 1)x + (Y − 1)y

d_x,y_.dist = infinity

CG4: Initial raster (D) resolution 500 × 500 m (i.e., 4 cells = 1 km^2^)

CG9: D resolution 333.3 × 333.3 m

CG16: D resolution 250 × 250 m

# Choose the centre cells for the aggregation process

For i = 1..25361:

x = random_integer in range(1,X) inc. end points

y = random_integer in range(1,Y) inc. end points

d_x,y_.belongs_to = d_x,y_.id

d_x,y_.dist = 0

chosen_list.append(d_x,y_)

# Find which centre cell is closest to every other cell in D

For all cells c in chosen_list:

For all cells d in D but not in chosen_list:

dist = sqrt((c_x_ - d_x_)^2^ + (c_y_ - d_y_)^2^)

if dist < d_x,y_.dist:

d_x,y_.belongs_to = c.id

if dist = = d_x,y_.dist:

if random_in_[0,1] < 0.5:

d_x,y_.belongs_to = c.id

# Dissolve the grid D according to the .belongs_to attrib ute

## Supporting Information

Figure S1 Population Distributions after Random Movement in Irregular Virtual Landscapes A matrix showing population distributions after a number of random movement scenarios in irregular landscapes. Rows (from top to bottom): 1) CGD4; 2) CGD9; 3) CGD16; 4) Dirichlet; and 5) aggregate map. Columns (from left to right): (A) random movement with *t* = 5 (time steps) and *p* = 1 (probability of movement in a time step); (B) random movement, *t* = 100, *p* = 1; and (C) directed random movement, *t* = 50, *p* = 1.
(8.9 MB PDF)

## Abbreviations

BNG: British National Grid
CGD: coarse-grain Dirichlet
UK: United Kingdom
