# Supplementary figures and images for: Landscape as a Model: The Importance of Geometry
Source: PLoS Comput Biol. 2007 Oct 26;3(10):e200. doi: 10.1371/journal.pcbi.0030200 (PMC2041976; doi:10.1371/journal.pcbi.0030200)

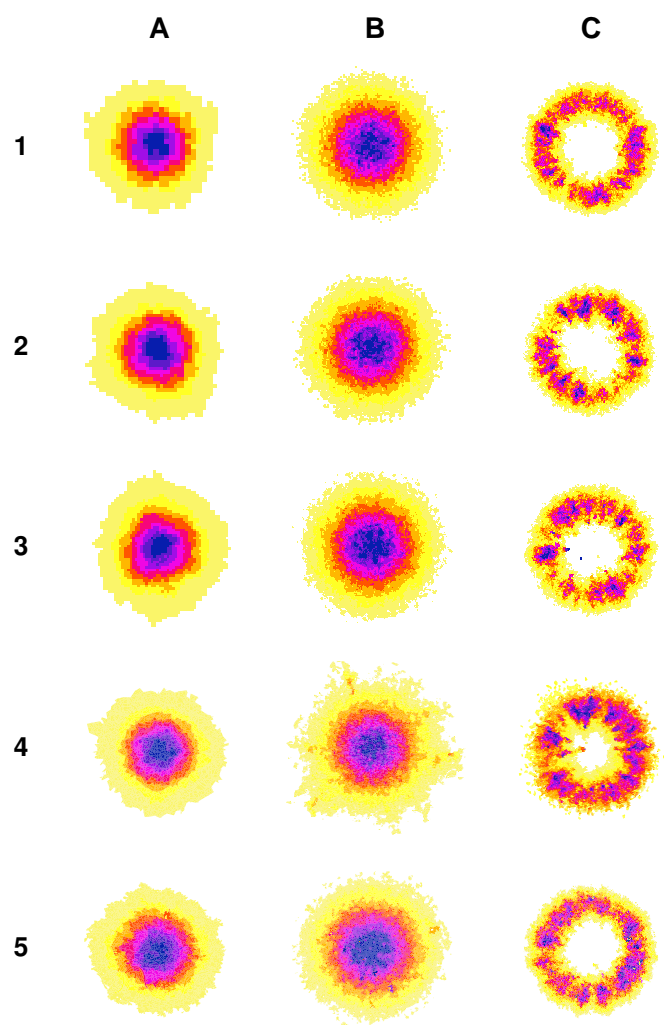

**FIGURE S1 POPULATION DISTRIBUTIONS AFTER RANDOM  
MOVEMENT IN IRREGULAR VIRTUAL LANDSCAPES**

Supplement: Figure S1 — A matrix showing population distributions after a number of random movement scenarios in irregular landscapes. Rows (from top to bottom): 1) CGD4; 2) CGD9; 3) CGD16; 4) Dirichlet; and 5) aggregate map. Columns (from left to right): (A) random movement with t = 5 (time steps) and p = 1 (probability of movement in a time step); (B) random movement, t = 100, p = 1; and (C) directed random movement, t = 50, p = 1. (8.9 MB PDF) [file pcbi.0030200.sg001.pdf]
